# Necrotizing Periodontal Diseases in a Semirural District of South Africa

**DOI:** 10.1155/2011/638584

**Published:** 2011-06-21

**Authors:** Neil Hamilton Wood, Elaine Blignaut, Johan Lemmer, Robin Meyerov, Liviu Feller

**Affiliations:** ^1^Department of Periodontology and Oral Medicine, School of Oral Health Sciences, Faculty of Health Sciences, University of Limpopo, Box D 26, Medunsa 0204, South Africa; ^2^Faculty of Dentistry, University of Sydney, Sydney, NSW 2010, Australia

## Abstract

*Objectives*. The aim of this study was to characterize the lesions of necrotizing gingivitis (NG) and necrotizing periodontitis (NP) with regard to extent and severity, and to correlate these parameters with the host HIV serostatus, CD4+ T-cell count, neutrophil count, age, and gender. *Methods*. Eighty-four consecutive patients, 39 black females and 45 black males aged 20–46 years, diagnosed with NG/NP were recruited to the study over a period of two years. *Results*. For both HIV-seropositive and -seronegative patients, the mandibular anterior gingiva was most frequently affected; 74% had NG/NP affecting ≥5 gingival tooth sites. Ninety percent of all patients had a mean severity of ≤4 mm. There was no statistically significant association between either extent or severity of NG/NP and HIV serostatus, CD4+ T-cell count, neutrophil count, age, or gender. The difference between the number of HIV-seropositive patients with NG/NP who had CD4+ T-cell counts ≤200 cells/mm^3^ and those who had CD4+ T cell counts of 201–499 cells/mm^3^ was not statistically significant. *Conclusion*. The clinical signs of NG/NP are similar in HIV-seropositive and -seronegative patients, and are not related to CD4+ T-cell count, to neutrophil count, to gender, or to age.

## 1. Introduction

Necrotizing periodontal diseases, comprising necrotizing gingivitis and necrotizing periodontitis, is a distinct disease entity [1]. In this paper the terms necrotizing gingivitis (NG) and necrotizing periodontitis (NP) are used in preference to the usual terms necrotizing ulcerative gingivitis and necrotizing ulcerative periodontitis. In NG the ulceration is not a primary feature but is secondary to the marginal gingival necrosis and in NP the necrosis affects the periodontal attachment apparatus, so the term ulceration is inappropriate.

Necrotizing gingivitis is characterised by marginal gingival necrosis starting at the tip of the interdental papilla, by gingival bleeding and by pain with the subsidiary features of pseudomembrane formation, cervical lymphadenopathy and foetid breath [1–3]. Necrotizing periodontitis is not a primary disease. It is an extension of the disease process into the periodontal attachment apparatus. Necrotizing gingivitis is common; NP is uncommon.

The pathogenesis of NG is multifactorial and involves a complex interaction between infection of the gingiva by periodontopathic bacteria, mainly spirochetes and fusiform bacilli, a susceptible host, disease-modulating factors such as immune suppression, malnutrition and other debilitating conditions, and factors deregulating local immune responses such as tobacco smoking, candida, and herpesviruses in the dentogingival plaque [4–9].

Necrotizing gingivitis and NP are more frequent in HIV-seropositive than in HIV-seronegative patients, but the dentogingival periodontopathic microbial flora, the clinical manifestation, the course of the disease, and the response to treatment in both groups are similar [7, 10]. Necrotizing gingivitis is more prevalent in HIV-seropositive patients with early HIV infection than in those with AIDS [11] and may be the first clinical indicator of HIV infection [12].

The purpose of this study was to characterise the lesions of NG/NP with regard to extent and severity, and to correlate these parameters with the host HIV serostatus, CD4+ T-cell count, neutrophil count, age and gender.

## 2. Material and Methods

### 2.1. Subject Population

Approval for this study was granted by the Research and Ethics Committee of the University of Limpopo, Medunsa Campus. Eighty-four patients from the Ga-Rankuwa district in South Africa diagnosed with NG/NP in the Department of Periodontology and Oral Medicine were recruited over a period of two years and the purpose of the study was explained to them. There were 39 females and 45 males. All were black persons between the ages of 20 and 46 years and all were of a similar socioeconomic status. None of the participants knew their HIV serostatus, and none were taking antiretroviral therapy at the time of NG/NP diagnosis. 

Of the 84 participants, 54 gave informed consent for HIV testing. The HIV serostatus of these patients was determined by enzyme-linked immunosorbent assay (ELISA) and Western blot. CD4+ T-cell counts were determined for 55 of the 84 participants, and neutrophil counts for 52. CD4+ T-cell counts were stratified into 3 groups: ≤200 cells/mm^3^, 201–499 cells/mm^3^, ≥500 cells/mm^3^. Neutropenia was defined as a neutrophil count less than 2000 cells/mm^3^, and the neutrophil counts were stratified into two groups: >2000 cells/mm^3^ (normal), and ≤2000 cells/mm^3^ (neutropenia).

The participants who agreed to be tested for HIV infection were counselled by a designated health care worker prior to the HIV testing and again when they were informed of the results. Participants who were found to be HIV-seropositive were referred immediately to the regional HIV clinic for treatment.

### 2.2. Periodontal Health Status

In this study all cases of NG/NP were diagnosed, and clinical information necessary for this study was collected by a single specialist in Oral Medicine and Periodontology (NHW). Necrotizing gingivitis was diagnosed by marginal gingival necrosis, gingival bleeding and pain, and necrotizing periodontitis by extension of this disease process into the periodontal attachment apparatus [1].

Data recording was carried out as has been described elsewhere [7], and each data set was given a unique number for confidentiality. Necrotizing gingivitis and NP were grouped together. A single site of NG/NP was defined as the disease related to the buccal or palatal/lingual gingiva of a single tooth. If an interdental papilla was involved, then two sites would be recorded as being affected. 

Severity at any site was determined by measuring the vertical width of the visibly necrotic gingiva with a periodontal probe. The severity was classified as being >4 mm of gingival necrosis or ≤4 mm of gingival necrosis. The extent of the lesions in any patient was classified according to the number of gingival tooth sites affected: those having ≥5 NG/NP sites and those with <5 NG/NP sites.

All patients received the standard treatment for NG/NP as has been described previously [9, 12]: a 5-day course of metronidazole 400 mg 3 times per day, paracetamol 500 mg 3 times per day, and chlorhexidine digluconate 0,2% mouthwash twice daily, and after this 5-day regimen plaque control instruction, scaling, and if necessary root planing were carried out.

### 2.3. Statistical Analysis

All data were entered in Microsoft Excel and all statistical analyses were performed on SAS, running under Microsoft Windows Vista Business for a personal computer. Analysis of variance (ANOVA), *t*-tests, Fisher's exact tests, and a logistic regression analysis were performed. *P* values of <.05 were regarded as statistically significant.

## 3. Results

The demographic data of all participants are shown in Table 1. There were 84 patients (mean age 30 years) with NG/NP, 45 males (54%), and 39 females (46%). Nine of the patients were tobacco smokers (11%). Thirty participants refused HIV testing (36%) and their HIV serostatus remained unknown. Of the 54 participants who agreed to be tested, 43 were HIV seropositive (80%) and 11 (20%) were HIV-seronegative. The mean age of 26 years of the HIV-seronegative patients with NG/NP was significantly lower than the mean age of 31 years of HIV-seropositive patients with NG/NP (pairwise *t*-tests,  *P* = .05). Of the HIV-seropositive group of 43 patients with NG/NP, 27 were female (63%) and 16 were male (37%) (Table 1).

Neutrophil counts were available for 52 patients; 36 (69%) had a normal neutrophil count and 16 (31%) had neutropenia. The HIV serostatus was known only for 47 of these 52 patients: 37 were HIV-seropositive and 10-seronegative. Ten of the 37 HIV seropositive patients (27%) had neutropenia and 4 of the 10 HIV-seronegative patients (40%) had neutropenia. 

CD4+ T-cell counts were available for 55 of the patients, for 49 of whom the HIV serostatus was known: 39 were HIV seropositive and 10 were HIV seronegative (Table 2). There was no statistical significance between the number of patients with CD4+ T-cell counts ≤200 cells/mm^3^ and the number of patients with CD4+ T-cell counts of 201–499 cells/mm^3^ (Fischer's exact test *P* = .16). 

The mandibular anterior sextant was the most frequently affected sextant (Tables 3(a)-3(c)). 

Data for frequency of tooth sites affected were available for 78 of the 84 patients. Fifty-eight patients (74%) had NG/NP affecting ≥5 tooth sites and 20 patients (26%) had <5 tooth sites affected (Table 4). There were statistically significantly more HIV-seropositive patients with ≥5 tooth sites affected than there were with <5 tooth sites affected (*z*-test, P<.05). There was an equal number of HIV-seronegative patients with NG/NP to the extent of ≥5 tooth sites and <5 tooth sites affected (Table 4).

When evaluating all 776 NG/NP tooth site lesions in all HIV-seropositive patients, HIV-seronegative patients, and those with unknown HIV serostatus together, 87 individual tooth site lesions (11.3%) had a severity of >4 mm and 689 (88.77%) a severity of  ≤4 mm. This same trend was observed when the severity of all NG/NP sites was evaluated for the 38 HIV-seropositive patients in whom 8% of tooth site lesions (31/402) were >4 mm, and 92% (371/402) were ≤4 mm.

There was no statistically significant relationship between either extent or severity on the one hand, and HIV serostatus, CD4+ T-cell count, neutrophil count, age, or gender on the other (logistical regression analysis).

## 4. Discussion

Necrotizing gingivitis is a disease of young adults and rarely occurs before adolescence [13]. In this study the mean age of the participants was 30 years. The mean age of the HIV-seropositive patients with NG/NP (31 years) was statistically significantly higher than that of HIV-seronegative patients (26) with NG/NP (pairwise *t*-test, *P* = .05) (Table 1). This finding is difficult to explain. 

In our study, as in previously published data [7], in both HIV-seropositive and -seronegative patients the gingiva of the mandibular anterior sextant was most frequently affected by NG/NP (Tables 3(a)-3(c)). For both HIV-seropositive and -seronegative groups, there were more patients in whom NG/NP affected ≥5 tooth sites than  <5 tooth sites, but there were equal numbers of HIV-seropositive patients who had <5 and ≥5 tooth sites affected (Table 4). There were more NG/NP lesions which had a severity of ≤4 mm than >4 mm (Table 4). For both extent and severity, these findings are in line with other reported data [14].

With current knowledge of the aetiopathogenesis of NG/NP, the tissue necrosis in NG/NP cannot be explained, nor can it be explained why the gingiva of anterior mandibular teeth is most affected, or why in some patients the NG/NP is widespread while in other patients it is confined to only a few teeth. 

A number of studies have shown that NG/NP is more prevalent in HIV-seropositive than in HIV-seronegative immunocompetent patients [11]. There are several reasons for this difference. Firstly, HIV-induced systemic immune suppression and HIV-associated malnutrition may lead to increased susceptibility to NG/NP [9]. Secondly, the dentogingival plaque of HIV-seropositive patients is known to harbour herpesviruses and candida species that can contribute to deregulation of local immune and inflammatory responses, thus promoting the development of NG/NP [9, 15, 16].

It is possible that HIV-associated cytokine dysregulation and qualitative defects of neutrophils, macrophages and Langerhans cells in the gingival tissues may play an important role in the pathogenesis of NG/NP and may be responsible for the increased frequency of this disease in HIV-seropositive patients compared to HIV-seronegative patients. 

Our case series comprised patients with NG/NP none of whom knew their own HIV serostatus. Despite the fact that 80% of those who consented to HIV testing were found to be HIV seropositive which is certainly a striking observation, the design of our study does not permit us to draw any conclusions from this regarding NG/NP being an indicator for HIV infection in patients with unknown HIV serostatus. However, the prudent clinician will be advised by this observation.

There was no statistically significant difference between the number of HIV-seropositive patients with NG/NP with CD4+ T-cell counts of ≤200 cells/mm^3^ and the number of those with CD4+ T-cell counts of 201–499 cells/mm^3^ (*P* = .34). Neutrophil counts were available for 52 patients: neutrophil counts of 36 patients (69%) were within the normal range, and 16 patients (31%) were neutropenic. There was no statistically significant association between extent or severity of NG/NP on the one hand, and age, gender, HIV serostatus, CD4+ T-cell count, or neutrophil count on the other.

Thirty of the total of 84 participants (36%) refused to be tested for HIV infection (Table 1) in spite of having been informed about the reported association between NG/NP and HIV infection. Although antiretroviral treatment is available and affordable in the Ga-Rankuwa area in South Africa many patients chose not to be tested even after counselling by a healthcare professional. This could possibly be because of a perceived stigma related to HIV infection and fear of being shunned by their communities. The social and health concerns arising from this are difficult to address.

The treatment protocol that we advocate has proved in practice to be successful in both HIV-seropositive and -seronegative patients [9, 12, 14]. It avoids the discomfort and messiness of scaling and root planing in the presence of extensive acute gingival inflammation, and it is less liable to result in loss of periodontal attachment.

Once the inflammation and acute pain of NG have been controlled with systemic metronidazole, further management of the gingival condition becomes much easier for the dentist and less intimidating for the patient.

In our study, for logistical reasons related to long distances and patient finances, patient recall information was incomplete, or in the case of follow-up information obtained by telephone, probably unreliable. It was therefore not possible to draw any statistically valid conclusions as to the rate of recurrence. However, what information could be obtained suggests that the rate of recurrence was low.

## 5. Conclusion

Necrotizing gingivitis and necrotizing periodontitis more frequently affect the anterior mandibular gingiva than other sextants. NG/NP generally affects the gingiva of  ≥5 teeth and typically each affected site has ≤4 mm of necrotic gingiva. The clinical signs are similar in HIV-seropositive and -seronegative patients, and are not related to CD4+ T-cell count, to neutrophil count, to gender, or to age.

The mean age of HIV seronegative patients with NG/NP is statistically higher than that of HIV-seropositive patients with NG/NP, but there is no statistically significant association between extent or severity of NG/NP on the one hand, and age, gender, HIV-serostatus, CD4+ T-cell count, or neutrophil count on the other.

## Figures and Tables

**Table 1:** Demographic data of the study population.

	HIV positive	HIV negative	Unknown	Total
*Gender*				
Males	16 (19%)	8 (10%)	21 (25%)	45 (54%)
Females	27 (32%)	3 (4%)	9 (11%)	39 (46%)
Total	43 (51%)	11 (14%)	30 (36%)	84 (100%)
*Smokers*	0	2	7	9
*Age*				
Mean	31	26	30	30
Standard deviation	*5.848*	*5.899*	*5.3*	*5.859*

**Table 2:** The CD4+ T-cell counts of patients with NG/NP.

CD4+ T-cell count HIV serostatus	≤200	201–499	≥500	Total
No. of HIV-	24	10	5	39
seropositive patients with NG/NP				
No. of HIV-				
seronegative patients	—	1	9	10
with NG/NP				
No. of patients				
with NG/NP with	2	3	1	6
unknown HIV serostatus				

Total	26	14	15	55

**Table:** (a) The number of times specific sextants were affected with NG/NP in HIV-seropositive and -seronegative patients.

Sextant	Upper right posterior	Upper anterior	Upper left posterior
	14	18	18
	22	36	22

Sextant	Lower right posterior	Lower anterior	Lower left posterior

**Table:** (b) The number of times specific sextants were affected with NG/NP in HIV-seropositive patients.

Sextant	Upper right posterior	Upper anterior	Upper left posterior
	10	15	16
	21	30	20

Sextant	Lower right posterior	Lower anterior	Lower left posterior

**Table:** (c) The number of times specific sextants were affected with NG/NP in HIV-seronegative patients.

Sextant	Upper right posterior	Upper anterior	Upper left posterior
	4	3	2
	1	6	2

Sextant	Lower right posterior	Lower anterior	Lower left posterior

**Table 4:** The severity and extent of NG/NP in relation to the patients' HIV serostatus.

		Severity		Extent (gingival tooth sites)		Not documented
No. of	HIV status	≤4 mm	>4 mm	< 5	≥5	
patients	Positive	46	2	10	28	5
with	Negative	8	2	5	5	1
NG/NP	Unknown	26	4	5	25	0

Total		70	8	20	58	6
